# We shall overcome (drug resistance) some day

**DOI:** 10.18632/oncotarget.26550

**Published:** 2019-01-04

**Authors:** Geeta G. Sharma, Luca Mologni

**Affiliations:** Department of Medicine and Surgery, University of Milano-Bicocca, Monza, MB, Italy

**Keywords:** TKIs, ALK, lorlatinib, resistance, combination therapies

The discovery of the involvement of defined molecular pathway(s) in tumor development and therapeutic targeting of such pathway(s) has revolutionised cancer treatment. More than 50 targeted therapeutic agents have been approved by the Food and Drug Administration (FDA) in the last two decades [1]. Unlike traditional cytotoxic treatments, targeted therapies rely on the dominant gene aberration that drives tumor growth and target only affected/malignant cells while sparing the healthy/non-malignant cells. However, although targeted therapies are highly effective and have improved patients survival, they are rarely curative [2]. In most cases, resistance against targeted therapies arises sooner or later. We now know that most tumors employ a highly complex and interconnected network of genetic alterations to drive their malignant growth and sustain survival, hence, single agent targeted therapies often result into modest long-term effects [3]. A number of mechanisms have been identified that mediate acquired (over time) resistance against the targeted therapies, such as point mutations that alter the binding of the drug to its target, activation of additional parallel or downstream signalling pathways (oncogene independent) as well as lineage changes of the tumor cells. Conversely, why some patients do not respond to the targeted therapies from the beginning (intrinsic resistance) is yet to be understood.

**Figure 1:**
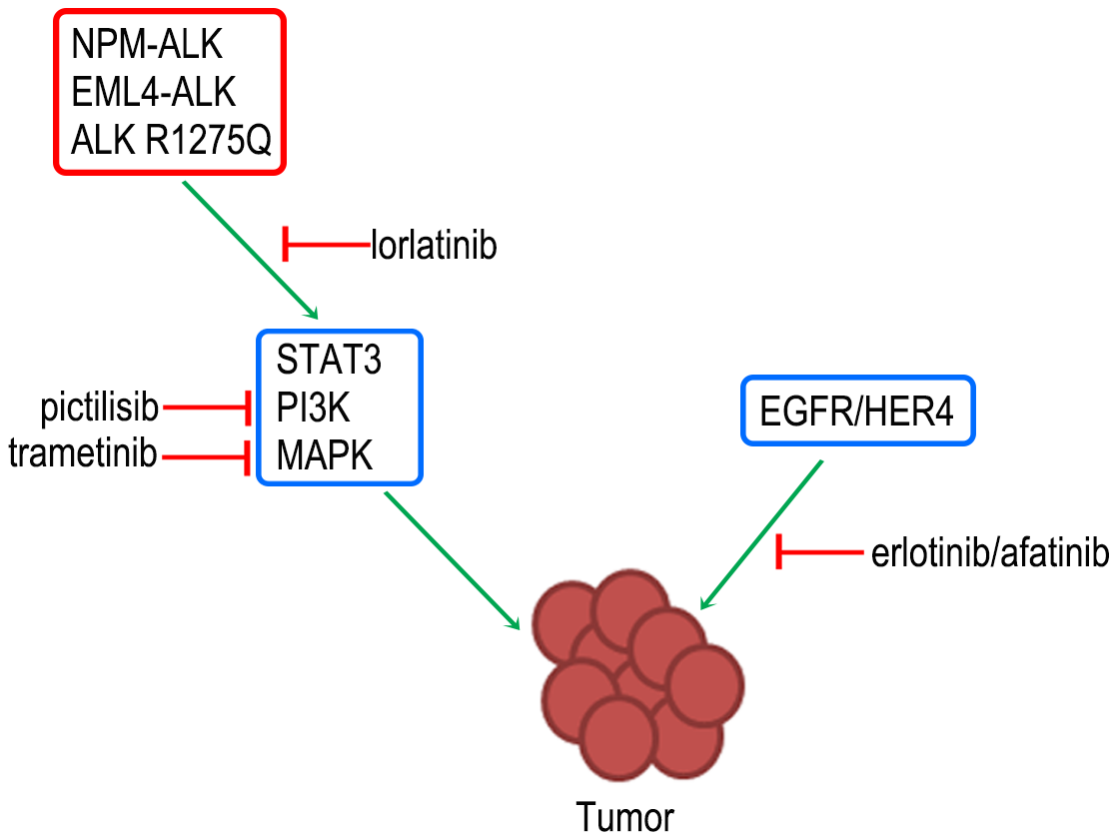
Use of combination therapy to target resistant tumor cells in ALK-positive cancers Off-target resistance mechanisms generally involve activation of bypass signalling pathways (blue boxes) which could be downstream of the oncogenic driver pathway (red box) or a parallel signalling pathway that could sustain survival and proliferation of tumor cells even in the presence of the TKI. This off-target resistance could be overcome by using a combination of lorlatinib treatment with other inhibitors such as pictilisib (PI3K inhibitor), trametinib (MAPK inhibitor), erlotinib (EGFR inhibitor) or afatinib (HER4 inhibitor) based on the involvement of the respective signalling pathways.

Anaplastic Lymphoma Kinase (ALK) exemplifies the use of targeted therapeutic agents in combating cancer, as well as the challenges that arise in terms of resistance mechanisms against such agents in clinical settings [4]. A number of ALK tyrosine kinase inhibitors (TKIs) have been developed and approved for use in ALK+ non-small cell lung cancer (NSCLC) while many are being evaluated in clinical trials for their activity in malignancies other than NSCLC [5]. The latest addition to the class of ALK inhibitor is lorlatinib (PF-06463922) which recently received Breakthrough Therapy Designation from the FDA. Besides improved potency and antitumor efficacy, lorlatinib has also been shown to inhibit one of the most resistant mutants, the solvent-front *ALK* G1202R mutation. It is also highly permeable through the blood brain barrier and hence effective in targeting central nervous system metastasis. Recently published results of the global phase II study has shown superior objective response rate (ORR) and intracranial ORR in comparison to the first and second generation ALK TKIs [6]. As the trends suggest, despite the potency and efficacy, resistance can be expected against lorlatinib treatment. In fact, the first case of resistance to lorlatinib was reported during its early-phase clinical testing where a double ALK mutation (C1556Y/L1198F) was detected in an ALK-positive NSCLC patient [7]. Organically, it became interesting to investigate the resistance mechanisms that could potentially arise against lorlatinib. Two studies were published this year shedding a light on the emergence of resistant mutations upon treatment with lorlatinib [8, 9]. One of the main findings of these two studies was that compound rather than single on-target resistant point mutations arise against lorlatinib. Another interesting finding in our case was the different ways different tumors acquired resistance. While ALK-positive anaplastic large cell lymphoma xenografts developed both on- and off-target mutations, NSCLC and neuroblastoma cells only showed off-target resistance mechanisms. Several cells that acquired resistance under lorlatinib treatment had alterations in different signalling pathways such as the PI3K/AKT/mTOR, RAS/MAPK and EGFR/ERBB4 signalling contributing to the resistant phenotype (Figure 1). Altogether, the findings point to the idea that with potent inhibitors, complex resistance mechanisms are likely to develop and monotherapies might not be adequate to improve overall survival in patients. We might be getting closer to target resistant tumor cells but we are still far from curing it. Given the complexity and multifaceted resistance mechanisms that tumor cells employ to evade therapies, perhaps, combination therapies are the future albeit not an easy one. Combination therapies have already shown to improve ORR as well as progression free survival in metastatic resistant BRAF^V600E/K^ melanoma patients that relapse on monotherapy [10]. However, in order to implement these combination therapies in ALK-positive cancers, it will be crucial to better understand the resistance mechanisms and their interconnections, as well as possible additional vulnerabilities of cancer cells, which would help in developing effective combined therapeutic strategies for cancer patients.
